# Spiritual Healing: A Triple Scoping Review of the Impact of Spirituality on Burn Injuries, Wounds, and Critical Care

**DOI:** 10.3390/ebj3010016

**Published:** 2022-02-24

**Authors:** Tomer Lagziel, Malik Muhammad Sohail, Harold G. Koenig, Jeffrey E. Janis, Stephen J. Poteet, Kimberly H. Khoo, Julie A. Caffrey, Sheera F. Lerman, Charles S. Hultman

**Affiliations:** 1 Department of Plastic and Reconstructive Surgery, Johns Hopkins University School of Medicine, Baltimore, MD 21224, USA; kkhoo2@jhu.edu (K.H.K.); jcaffre5@jhmi.edu (J.A.C.); chultma1@jhmi.edu (C.S.H.); 2 The Center for Health Policy & Inequalities Research, Duke University School of Medicine, Durham, NC 27710, USA; malik.sohail@duke.edu (M.M.S.); harold.koenig@duke.edu (H.G.K.); 3 Department of Plastic and Reconstructive Surgery, Ohio State University, Columbus, OH 43212, USA; jeffrey.janis@osumc.edu; 4 Ohio Plastic and Reconstructive Surgery, Memorial Hospital, Marysville, OH 43040, USA; stephen.poteet@osumc.edu; 5 Department of Psychiatry, Johns Hopkins University School of Medicine, Baltimore, MD 21205, USA; szohar1@jhmi.edu

**Keywords:** burn, spirituality, complimentary medicine, wounds, critical care

## Abstract

Burn patients are unique because their recovery requires prolonged hospital admissions, often complicated by a myriad of medical and surgical complications as well as psychological and emotional challenges. Religion and spirituality have been linked to improved health outcomes in other medical fields. Our scoping review aimed to examine the available literature for evidence of the impact of spirituality on burns, complex wounds, and critical care to shed more light on the relationship between spirituality and the conditions treated by multidisciplinary burn center teams. We performed three systematic reviews to examine the relationship between spirituality and these conditions. Searches were performed using MeSH terms utilizing four databases (MEDLINE via PubMed, Embase, Cochrane, Web of Science, and Scopus). A systematic and independent title/abstract screening was carried out by two independent reviewers and a full-text review was followed. Our review demonstrated a clear lack of overlap between study outcomes and lack of objective spirituality measurements. Most articles primarily focused on psychological outcomes, such as stress or mental health, instead of objective measures such as wound size or scar formation. We found a trend toward better psychological outcomes in patients with more spirituality, either pre-existing or interventional. To increase comparability and uniformity of outcomes, future studies would benefit from utilizing standardized spiritual assessment tools and objective wound metrics.

## 1. Introduction

In the past century, rapid technological advancements in medicine shifted care-oriented services to cure-oriented medical treatment, often resulting in the neglect of the spiritual dimension of health and illness [1]. Religion and spirituality have been recognized as significant contributing factors to health by numerous studies [1], with empirical evidence suggesting that people with higher levels of spirituality have lower levels of depression, improved quality of life, and higher pain tolerance [2].

The concepts of faith, spirituality, and religion are commonly understood in similar meaning; however, the concept of spirituality is distinct and unique in conceptualization and operationalization [3]. Religion is often viewed as more institutionalized, involves accountability, and is deeply rooted in tradition while spirituality is more personal and requires fewer obligations. Spirituality is an individual’s attempt to finding meaning in life and a sense of involvement with transcendence [4].

Patients suffering burn injuries may require a prolonged hospital stay with the utilization of critical care services, and burn injuries are unique as the nature of the injuries often causes psychological stress [5]. The specialized burn center teams that treat these injuries are also skilled to treat other complex non-burn wounds. There is substantial evidence that psychological stress adversely affects the immune system. Any form and level of stress can have significant consequences for wound healing [6], and empirical evidence shows that wound healing may take significantly longer for individuals who are stressed [7]. Additionally, delayed healing is associated with elevated anxiety and depression [8]. Considering the substantial level of evidence showing that negative psychological and emotional states have detrimental impacts on the process of wound healing and immune recovery, we aimed to understand the role that spirituality plays in mitigating these deleterious repercussions.

The purpose of our scoping review is to assess the current literature for reports on the impact of spirituality on burn injuries, complex wounds, and critical care recovery to analyze the relationship between spirituality and the conditions treated by multidisciplinary burn center teams. Specifically, in patients suffering from burn injuries, complex wounds, or those requiring critical care services what is the effect of spirituality on healing outcomes. These topics were chosen as they are within the direct treatment scope of multidisciplinary burn center teams.

## 2. Materials and Methods

For this scoping review, we followed the Preferred Reporting Items for Systematic Reviews and Meta-Analysis, Scoping Review extension (PRISMA-ScR) guidelines (http://www.prisma-statement.org/Extensions/ScopingReviews) (accessed on 23 February 2022), and we did not register our search protocol before this publication. We performed three systematic reviews to examine the relationship between spirituality and each of the three injury classes: burns, wounds, and critical care. The intention was to prepare reports first from each of the three systematic reviews separately and then synthesize findings altogether. However, due to the limited literature regarding this specific topic area, there was insufficient empirical data available for quantitative synthesis. As such, we report the studies’ findings from the individual systematic reviews as the authors have published them and synthesized them based on how spirituality was determined and what outcome was assessed.

### 2.1. Eligibility Criteria

We employed the participants, interventions, comparisons, outcomes, and study design (PICOS) strategy for study inclusion during the selection process for all three reviews. For the analysis, participants were at least 18 years of age, treated as an inpatient, suffered a burn-related injury (thermal, scald, contact, electrical, chemical) of any percent total body surface area (%TBSA), similar complex wound, and/or required inpatient critical care services (Table 1). Spirituality services had to be provided for these patients; we did not limit spirituality to religion—any study that aimed to assess spirituality, religious or otherwise, along with the above-mentioned concepts was deemed eligible for review. No comparisons were made between the three observation groups due to the limited quantitative evidence available. We observed the different surgical procedures, the type and size of the injuries sustained, and the different reasons for critical care services. Outcomes measured were length of stay, injury location, hospital-acquired complications, mortality. Study designs considered were randomized controlled trials (RCTs), non-randomized controlled trials, retrospective studies, observational studies, prospective studies, case-control studies, cohort studies, and surveys. There was no predetermined length of participant follow-up. Studies were excluded if they were not in English; were published before the year 2000; were review articles without new academic contribution, non-reviewed peer literature, case reports, editorial articles, unavailable full-text; or patient death occurred before hospital admission.

### 2.2. Information Sources

Three preliminary literature searches were performed using MeSH terms, by our medical library informationist (S.M.S.), utilizing five databases (MEDLINE via PubMed, Embase, Cochrane, Web of Science, and Scopus) with no starting limit through 31 October 2021. Detailed search strategies can be found in Supplementary Material S1. A list of references for relevant articles was compiled and hand-searched to identify additional applicable studies. All references were imported into Covidence (Veritas Health Innovation Ltd., Melbourne, Australia) reference management software for proper data extraction and removal of duplicate articles.

### 2.3. Selection of Sources and Evidence

A systematic and independent title/abstract screening was carried out by two independent reviewers (T.L. and M.M.S.) and a full-text review was followed to guarantee quality and accuracy during the process. Any differences in opinion involving inclusion or exclusion of a particular study were discussed and resolved between the two reviewers. Any further conflict or disagreement that persisted after the discussion was resolved by a third reviewer (C.S.H.). The following data points were extracted qualitatively and quantitatively: authors, year of publication, type of study, sample size, male and female distributions, age, spirituality services provided, injury characteristics, hospital LOS, hospital-acquired complications, and mortality. To avoid any duplicate reporting of results, one data collection form was used in cases where one study provided multiple reports.

### 2.4. Synthesis of Results

We grouped the studies by the types of medical condition analyzed, and summarized the type of spiritual intervention assessed, clinical outcomes observed, and study designs for each group. The number of patients observed in each study was recorded along with whatever significant findings were reported.

### 2.5. Analysis Discrepancies

Spirituality was the only common primary variable across the three reviews. However, there was no uniform definition of spirituality utilized across all. Furthermore, most studies did not assess the same outcomes. For studies that reported empiric data, sample sizes across the studies were small. Many studies that explored the topics of this review were qualitative in nature and did not allow for quantitative reporting in our review, but their findings are still discussed. Furthermore, we set out to analyze the impact of spirituality on length of stay, injury location, hospital-acquired complications, and mortality. However, we discovered that most articles did not discuss these outcomes, but we sought to report all outcomes reviewed.

## 3. Results

### 3.1. Selection of Sources of Evidence

#### 3.1.1. Spirituality and Burn Wounds

**Figure 1:**
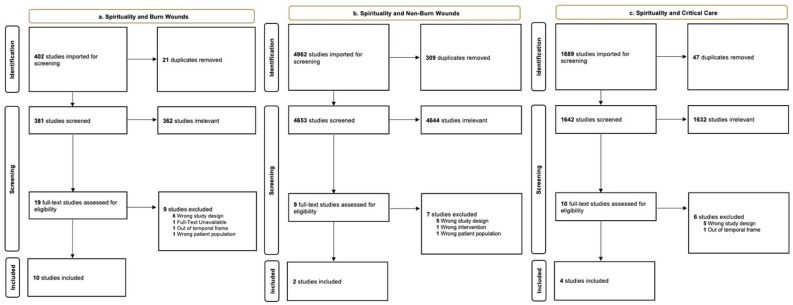
PRISMA flow chart describing the results of the final article selection and screening process: (**a**) burn injury and spirituality; (**b**) complex wounds and spirituality; (**c**) critical care and spirituality.

We screened 481 unique records from the following databases: PubMed, Embase, Cochrane, Web of Science, and Scopus. No additional records were identified through other sources. Title and abstract review identified a total of 16 studies for potential inclusion. After a full-text review, 10 studies met the eligibility criteria and were included in the review (Figure 1a). The 10 studies involved 1080 burn patients and were published between 2002 and 2020. The studies included prospective and retrospective cohort research designs along with clinical trials and case series.

#### 3.1.2. Spirituality and Non-Burn Wounds

We screened 4653 unique records from the following databases: PubMed, Embase, Cochrane, Web of Science, and Scopus. No additional records were identified through other sources. Title and abstract review identified a total of nine studies for potential inclusion. After a full-text review, two studies met the eligibility criteria and were included in the review (Figure 1b). The two studies involved 51 patients with non-burn wounds and were published between 2004 and 2020. The studies contained retrospective research only.

#### 3.1.3. Spirituality and Critical Care

We screened 1642 unique records from the following databases: PubMed, Embase, Cochrane, Web of Science, and Scopus. No additional records were identified through other sources. Title and abstract review identified a total of 14 studies for potential inclusion. After a full-text review, two studies met the eligibility criteria and were included in the review (Figure 1c). The two studies involved 125 patients treated in the intensive care unit from non-wound and non-burn conditions which were published between 2017 and 2019. The studies included both prospective and retrospective research designs.

### 3.2. Combined Study Outcomes

#### 3.2.1. Spirituality and Burn Wounds

A primary observation from our review was a clear lack of overlap between study outcomes and lack of objective spirituality measurements. We found very little overlap in the outcome measures reported by the studies screened (Table 2). Additionally, most studies observed how spirituality impacts psychological and psychosocial recovery from burn injuries, rather than objective wound recovery metrics such was epithelization, wound size, discoloration, or scar formation. Two case series with similar spirituality assessments that focused on belief in a higher power or religious affiliation that was present before or after injury found improved resilience in patients with spiritual orientation [9,10]. The improved resilience was noted to allow patients’ better coping abilities with their post-injury lives. Providing a similar assessment, Ajoudani and colleagues found improved post-traumatic growth with patients that had a higher spirituality score [11]. We encountered two studies that assessed the relationship between spirituality and pain in burn patients. Dauber and colleagues performed a survey study with spirituality defined as a belief in “God” or practicing yoga which was correlated to improved pain. Similarly, Keivan and colleagues performed a RCT with controlled spiritual care sessions which resulted in improved pain scores and better reported pain control [12,13]. Nasiri and colleagues also reported reduced anxiety for these patients, a similar finding to those of Jibeen and colleagues who reported less psychological distress for patients with a higher Spiritual Transcendence Index [14,15]. Yoga was also mentioned as a form of spirituality that resulted in improved quality of life, self-esteem, and body image by two studies [16,17]. Hultman and colleagues found that patients with worse injury characteristics, such as higher %TBSA, a greater number of hospital-acquired complications, or a longer hospital stay, required significantly more chaplain visits but also found slightly lower, yet not statistically significant, mortality for patients with a religious affiliation [18].

#### 3.2.2. Spirituality and Non-Burn Wounds

Only two studies were found to examine healing after non-burn-related wounds and spirituality. Neither study was able to provide statistically significant relationships, as they were limited by smaller sample sizes. One study was comprised of a literature review and case series of wounds inflicted on patients practicing Sufism [19]. A second study assessed the relationship between spirituality and religiosity and coping with sports injuries, which were defined as any injury sustained during physical activity that impaired said activity [20]. Consistent with the findings of studies involving burns, religiosity and spirituality were noted to have a positive impact on coping efforts and injury management.

#### 3.2.3. Spirituality and Critical Care

Similar to the literature for non-burn wounds, few studies were found to examine the role of spirituality in the intensive care unit. Furthermore, most studies we found did not report statistically significant empirical evidence and had small sample sizes. However, a RCT by Yadak and colleagues examined the effects of Quran reciting on mechanical ventilation weaning in intensive care patients but did not find a statistically significant difference in respiratory-related physiological parameters [21]. While the direct relationship between spirituality and patient status was not directly assessed in their study, Swinton et al. reported on the importance of spirituality for the clinicians and family members [22].

## 4. Discussion

In the dictionary, spirituality is described as “…sensitivity or attachment to religious values” [23]. From a historical perspective, this relationship to religion holds true but the definition of spirituality has evolved in recent times [24]. In 2012, the International Consensus Conference defined spirituality as “a dynamic and intrinsic aspect of humanity through which persons seek ultimate meaning, purpose, and transcendence, and experience relationship to self, family, others, community, society, nature, and the significant or sacred” [25]. In medicine, spirituality or spiritual intervention have no clear definition, which carries a limitation for this review and future studies. Spirituality has been critically assessed in medical literature, particularly in the fields of oncology and palliative care [26,27]. However, there is a lack of appropriate literature that examines the relationship between spirituality to burn injuries, non-burn wounds, and critical care. We set out to analyze the impact of spirituality on burn wound healing.

Consistent with the findings in our review, studies from other medical fields reported that providing spiritual care showed improvements in outcomes, coping skills, and psychosocial well-being [28,29,30]. One paper suggested five potential mechanisms behind the salutogenic impact of faith on healing: “behavioral/conative”—faith as a facilitator for healthy behavior; “interpersonal”—faith as a facilitator for support systems; “cognitive”—establishing a positive mental framework; “affective”—provoking soothing emotions to combat stress; “psychophysiological”—providing a hopeful future to allow the current burden to be tolerated [31]. In our review, we also found an illustrative case report that suggested nurses integrate spirituality into their treatment of patients to provide more holistic care in the intensive care unit [32]. Though these theories and approaches may not demonstrate any direct evidence of the influence of spirituality on objective burn or wound outcomes, we see that spirituality improves psychological stress, which may potentially alleviate negative impacts of stress on the immune response.

Regarding the psychological impact of yoga, the only studies that measured this association were those studying burn injuries [12,16,17]. However, a RCT on surgical breast cancer patients reported significantly improved quantitative clinical outcomes in patients practicing yoga [33]. Though the literature defines yoga as a spiritual practice, yoga requires some level of physical fitness and activity. As such, it is impossible to differentiate if the improved outcomes in this reported study are due to spiritual or physical exercises.

Burn patients are unique because their recovery requires prolonged hospital admissions, often complicated by a myriad of medical and surgical complications as well as psychological and emotional challenges [34,35]. It has been suggested that spirituality can help patients manage their recovery more effectively [36,37], and our results from this scoping review support a beneficial relationship between spirituality and, at the very least, the psychological symptoms associated with burn injuries.

One limitation of this review is the inability to synthesize data collected from these three literature searches. The lack of literature examining the other conditions managed by multidisciplinary burn center teams (complex wounds and critical care) limited the power of a quantitative synthesis; however, the few reports that we found did suggest a beneficial relationship between spirituality and complex wounds or critical care, both for the patient and family.

Our study is also limited by the method of assessing spirituality among the different studies. Some studies call for interventional spirituality and assess clinical outcomes based on that. Other studies, conversely, rely on pre-existing spirituality as a sufficient variable in understanding the impact on clinical outcomes.

Another limitation encountered in this review was the lack of applicable studies discussing the association between spirituality and objective wound healing metrics. A majority of articles primarily focus on psychological outcomes, such as stress or mental health, instead of objective measures such as wound size or scar formation. Along the same lines, there was no single, uniform definition of “spirituality,” adding to the challenge of searching for studies that objectively measure the impact of spirituality on burn, wound, or critical care patients. More empirical research is needed with standardized definitions in order to draw clinically and statistically significant conclusions. To increase comparability and uniformity of outcomes, future studies would benefit from utilizing standardized spiritual assessment tools such as the Belief into Action (BIAc) scale, a tool developed to quantify religious behaviors, to report more objective results [38].

## 5. Conclusions

The complex nature of burn injuries requires specialized multidisciplinary care. Burn surgeons have treated patients suffering from burns and complex wounds, many times requiring critical care. Our review shows a trend toward better psychological outcomes in patients with more spirituality, either pre-existing or interventional. However, there is very little overlap between the different spiritual interventions and definitions as well as a lack of overlap in the study outcomes. This limited our ability to perform a meta-analysis and draw clinically or statistically significant conclusions. There exists a large gap in the literature that warrants further investigation. A future prospective RCT should be planned with a standardized spirituality definition to analyze how spirituality impacts burns, wounds, and critical care.

## Figures and Tables

**Table 1:** Inclusion and exclusion criteria.

Inclusion Criteria	Exclusion Criteria
≥18 Years of Age	Brain Trauma
Inpatient Treatment Setting	Death Prior to Admission
Spirituality Assessment/Intervention	Pediatric Studies
Burn Injury (Burn Review)	Animal Studies
Complex Wound (Wound Review)	Review Articles
Critical/Intensive Care (Critical Care Review)	Editorial/Commentary Articles
English Language	Publication Date <2000

**Table 2:** Combined study outcomes for studies assessing spirituality and burn injuries.

Author	Year	Design	Location of Study	No. of Patients	%TBSA (Range or Mean)	Spirituality Assessment	Improved Outcomes Reported
T.E. Abrams et al. [9]	2018	Case Series	USA	8	20–98	Higher Power Belief	• Resilience
N. Williams et al. [10]	2003	Case Series	USA	8	5–95	Religious Affiliation	• Resilience
F. Ajoudani et al. [11]	2019	Cross-Sectional Study	Iran	50	32.90 ± 6.11	Spiritual Well-Being Scale	• Post-traumatic growth
A. Dauber et al. [12]	2002	Survey	USA	358	38 ± 22.5	God, Yoga	• Pain
N. Keivan et al. [13]	2019	Randomized Control Trial	Iran	68	48.50 ± 17.20	Spiritual Care Sessions	• Pain
M. Nasiri et al. [14]	2020	Clinical Trial	Iran	71	29.8 ± 9	Reciting God’s Name	• Pain • Anxiety
T. Jibeen et al. [15]	2018	Cross-Sectional Study	Pakistan	98	36.9 ± 19.5	Spiritual Transcendence Index	• Psychological Distress • Behavioral Traits
C. Miller et al. [16]	2015	Case Control	USA	5	5–65	Yoga	• Range of Motion • Quality of Life
A. Ozdemir et al. [17]	2019	Prospective Cohort	Turkey	100	49.3 ± 13.1	Yoga	• Self-Esteem • Body Image
C.S. Hultman et al. [18]	2014	Retrospective Cohort	USA	314	11.8	Religious Affiliation	• Mortality

## Data Availability

All studies reviewed in this paper have been appropriately cited and are publicly accessible.

## Supplementary Materials

The following supporting information can be downloaded at: https://www.mdpi.com/article/10.3390/ebj3010016/s1, Supplementary Material S1: Database search summaries.
