# Supplementary material for: Spiritual Healing: A Triple Scoping Review of the Impact of Spirituality on Burn Injuries, Wounds, and Critical Care
Source: Eur Burn J. 2022 Feb 24;3(1):188–96. doi: 10.3390/ebj3010016 (PMC11575372; doi:10.3390/ebj3010016)
Supplement: Supplementary file 1 [file ebj-03-00016-s001.zip › ebj-1561811-supplementary.pdf]

european burn journal
MDPI

Supplementary Material S1: Search Summary.

Search Summary 1.

Topic	Spirituality and Burn Wounds and Healing
Reference Manager	
Timeline	
Restrictions or Limitations	
Key Articles	
Databases	PubMed, Embase, Cochrane, Web of Science, Scopus
Date Run	November 2, 2021
Total Number of Results	615
Number of Duplicates Removed	213
Remaining Number of Results	402
Search Prepared By	Stella M. Seal, MLS

PubMed

Search number	Query	Results
1	(("Religion"[Mesh]) OR "Spirituality"[Mesh]) OR "Spiritual Therapies"[Mesh] OR "Anthroposophy" [tw] OR "Buddhism" [tw] OR "Catholicism" [tw] OR "Christian Science" [tw] OR "Christianity" [tw] OR "Church of Jesus Christ of Latter-day Saints" [tw] OR "Confucianism" [tw] OR "Eastern Orthodoxy" [tw] OR "Ethic, Religious" [tw] OR "Exorcism" [tw] OR "Exorcisms" [tw] OR "Faith Healing" [tw] OR "Hinduism" [tw] OR "Islam" [tw] OR "Jehovah's Witnesses" [tw] OR "Judaism" [tw] OR "Magic" [tw] OR "African Traditional Medicine" [tw] OR "Meditation" [tw] OR "Mental Healing" [tw] OR "Mysticism" [tw] OR "Prayer" [tw] OR "Prayers" [tw] OR "Protestantism" [tw] OR "Radiesthesia" [tw] OR "Religion and Medicine" [tw] OR "Religion and Psychology" [tw] OR "Religion and Science" [tw] OR "Religion and Sex" [tw] OR "Religions" [tw] OR "Religious Belief" [tw] OR "Religious Beliefs" [tw] OR "Religious Ethics" [tw] OR "Religious Missions" [tw] OR "Religious Philosophies" [tw] OR "Saints" [tw] OR "Shamanism" [tw] OR "Spirit Possession" [tw] OR "Spiritual Healing" [tw] OR "Spiritual Healings" [tw] OR "Spiritualism" [tw] OR "Spiritualities " [tw] OR "spirituality" [tw] OR "Spirituality" [tw] OR "Theology" [tw] OR "Therapeutic Touch" [tw] OR "Witchcraft" [tw] OR "Yoga" [tw]	98,428
2	("Wound Healing"[Mesh]) OR "Regeneration"[Mesh] OR heal* [tw] OR regenerat* [tw]	4,619,633
3	Burns [mesh] OR burn [tw] OR burns [tw] OR burned [tw] OR burnt [tw]	86,557
4	#1 AND #2 AND #3	98

Embase

No.	Query	Results
#1	'religion'/exp OR 'spirituality'/exp OR 'spiritual therapies'/exp OR anthroposophy:ti,ab,kw OR buddhism:ti,ab,kw OR catholicism:ti,ab,kw OR 'christian science':ti,ab,kw OR christianity:ti,ab,kw OR 'church of jesus christ of latter-day saints':ti,ab,kw OR confucianism:ti,ab,kw OR 'eastern orthodoxy':ti,ab,kw OR 'religious ethic':ti,ab,kw OR exorcism:ti,ab,kw OR exorcisms:ti,ab,kw OR 'faith healing':ti,ab,kw OR hinduism:ti,ab,kw OR islam:ti,ab,kw OR 'jehovahs witnesses':ti,ab,kw OR judaism:ti,ab,kw OR magic:ti,ab,kw OR 'african traditional medicine':ti,ab,kw OR meditation:ti,ab,kw OR 'mental healing':ti,ab,kw OR mysticism:ti,ab,kw OR prayer:ti,ab,kw OR prayers:ti,ab,kw OR protestantism:ti,ab,kw OR radiesthesia:ti,ab,kw OR 'religion':ti,ab,kw OR religions:ti,ab,kw OR 'religious belief':ti,ab,kw OR 'religious beliefs':ti,ab,kw OR 'religious ethics':ti,ab,kw OR 'religious missions':ti,ab,kw OR	118079

	'religious philosophies':ti,ab,kw OR saints:ti,ab,kw OR shamanism:ti,ab,kw OR 'spirit possession':ti,ab,kw OR 'spiritual healing':ti,ab,kw OR 'spiritual healings':ti,ab,kw OR spiritualism:ti,ab,kw OR spiritualities:ti,ab,kw OR spirituality:ti,ab,kw OR theology:ti,ab,kw OR 'therapeutic touch':ti,ab,kw OR witchcraft:ti,ab,kw OR yoga:ti,ab,kw	
#2	'wound healing'/exp OR 'regeneration'/exp OR heal*:ti,ab,kw OR regenerat*:ti,ab,kw	5051635
#3	'burn'/exp OR 'burn patient'/exp OR 'burn':ti,ab,kw OR 'burns':ti,ab,kw OR 'burned':ti,ab,kw OR 'burnt':ti,ab,kw	123603
#4	#1 AND #2 AND #3	148

Cochrane

Search Name:

Date Run: 03/11/2021 10:40:13

Comment:

ID	Search	Hits
#1	(([mh Religion]) OR [mh Spirituality]) OR [mh "Spiritual Therapies"] OR Anthroposophy:ti,ab,kw OR Buddhism:ti,ab,kw OR Catholicism:ti,ab,kw OR "Christian Science":ti,ab,kw OR Christianity:ti,ab,kw OR "Church of Jesus Christ of Latter-day Saints":ti,ab,kw OR Confucianism:ti,ab,kw OR "Eastern Orthodoxy":ti,ab,kw OR "Ethic, Religious":ti,ab,kw OR Exorcism:ti,ab,kw OR Exorcisms:ti,ab,kw OR "Faith Healing":ti,ab,kw OR Hinduism:ti,ab,kw OR Islam:ti,ab,kw OR "Jehovah's Witnesses":ti,ab,kw OR Judaism:ti,ab,kw OR Magic:ti,ab,kw OR "African Traditional Medicine":ti,ab,kw OR Meditation:ti,ab,kw OR "Mental Healing":ti,ab,kw OR Mysticism:ti,ab,kw OR Prayer:ti,ab,kw OR Prayers:ti,ab,kw OR Protestantism:ti,ab,kw OR Radiesthesia:ti,ab,kw OR "Religion and Medicine":ti,ab,kw OR "Religion and Psychology":ti,ab,kw OR "Religion and Science":ti,ab,kw OR "Religion and Sex":ti,ab,kw OR Religions:ti,ab,kw OR "Religious Belief":ti,ab,kw OR "Religious Beliefs":ti,ab,kw OR "Religious Ethics":ti,ab,kw OR "Religious Missions":ti,ab,kw OR "Religious Philosophies":ti,ab,kw OR Saints:ti,ab,kw OR Shamanism:ti,ab,kw OR "Spirit Possession":ti,ab,kw OR "Spiritual Healing":ti,ab,kw OR "Spiritual Healings":ti,ab,kw OR Spiritualism:ti,ab,kw OR Spiritualities:ti,ab,kw OR spirituality:ti,ab,kw OR Spirituality:ti,ab,kw OR Theology:ti,ab,kw OR "Therapeutic Touch":ti,ab,kw OR Witchcraft:ti,ab,kw OR Yoga:ti,ab,kw	8352
#2	([mh "Wound Healing"]) OR [mh Regeneration] OR heal*:ti,ab,kw OR regenerat*:ti,ab,kw	418884
#3	([mh "burns"]) OR "burn" OR "burns" OR "burned" OR "burnt"	8422
#4	#1 AND #2 AND #3	20

Web of Science

TS=(Religion OR Spirituality OR "Spiritual Therapies" OR Anthroposophy OR Buddhism OR Catholicism OR "Christian Science" OR Christianity OR "Church of Jesus Christ of Latter-day Saints" OR Confucianism OR "Eastern Orthodoxy" OR "Ethic, Religious" OR Exorcism OR Exorcisms OR "Faith Healing" OR Hinduism OR Islam OR "Jehovah's Witnesses" OR Judaism OR Magic OR "African Traditional Medicine" OR Meditation OR "Mental Healing" OR Mysticism OR Prayer OR Prayers OR Protestantism OR radiesthesia OR "Religion" OR Religions OR "Religious Belief" OR "Religious Beliefs" OR "Religious Ethics" OR "Religious Missions" OR "Religious Philosophies" OR Saints OR Shamanism OR "Spirit Possession" OR "Spiritual Healing" OR "Spiritual Healings" OR Spiritualism OR Spiritualities OR spirituality OR Spirituality OR Theology OR "Therapeutic Touch" OR Witchcraft OR Yoga)

AND

TS=("wound healing" OR regenerat* OR heal*)
AND
TS=(burn OR burns OR burned OR burnt)
Searched 11/2/2021
115 results

Scopus

234 document results
(TITLE-ABS-KEY (religion OR spirituality OR "Spiritual Therapies" OR anthroposophy OR buddhism OR catholicism OR "Christian Science" OR christianity OR "Church of Jesus Christ of Latter-day Saints" OR confucianism OR "Eastern Orthodoxy" OR "religious ethic" OR exorcism OR exorcisms OR "Faith Healing" OR hinduism OR islam OR "Jehovahs Witnesses" OR judaism OR magic OR "African Traditional Medicine" OR meditation OR "Mental Healing" OR mysticism OR prayer OR prayers OR protestantism OR radiesthesia OR "Religion" OR religions OR "Religious Belief" OR "Religious Beliefs" OR "Religious Ethics" OR "Religious Missions" OR "Religious Philosophies" OR saints OR shamanism OR "Spirit Possession" OR "Spiritual Healing" OR "Spiritual Healings" OR spiritualism OR spiritualities OR spirituality OR spirituality OR theology OR "Therapeutic Touch" OR witchcraft OR yoga) AND TITLE-ABS-KEY (burn OR burns OR burned OR burnt) AND TITLE-ABS-KEY ("wound healing" OR heal* OR regenerat*))

2. Search Summary 2.

Topic	Spirituality and Wound Healing
Reference Manager	Covidence
Timeline	
Restrictions or Limitations	
Key Articles	
Databases	PubMed, Embase, Cochrane, Web of Science, Scopus
Date Run	November 2, 2021
Total Number of Results	8139
Number of Duplicates Removed	3177
Remaining Number of Results	4962
Search Prepared By	Stella M. Seal, MLS

PubMed

Search number	Query	Results
1	((((((((((((((("Wounds and Injuries"[Mesh:NoExp]) OR "Accidental Injuries"[Mesh]) OR "Athletic Injuries"[Mesh]) OR "Barotrauma"[Mesh]) OR "Bites and Stings"[Mesh]) OR "Cold Injury"[Mesh]) OR "Fractures, Open"[Mesh]) OR "Frostbite"[Mesh]) OR "Lacerations"[Mesh]) OR "Microtrauma, Physical"[Mesh]) OR "Multiple Trauma"[Mesh]) OR "Radiodermatitis"[Mesh]) OR "Self Mutilation"[Mesh]) OR "Soft Tissue Injuries"[Mesh]) OR "Surgical Wound"[Mesh]) OR "War-Related Injuries"[Mesh]) OR "Wounds, Penetrating"[Mesh] OR "Accident Injuries" [tw] OR "Accident Injury" [tw] OR "Accidental Injury" [tw] OR "Athletic Injury" [tw] OR "Barotraumas" [tw] OR "Bite" [tw] OR "Bites" [tw] OR "Blast injuries" [tw] OR "Cold Injuries" [tw] OR "Compound Fracture" [tw] OR	1,665,199

	"Compound Fractures" [tw] OR "Decapitation" [tw] OR "Degloving injuries" [tw] OR "Frostbite" [tw] OR "Injuries" [tw] OR "Injuries and Wounds" [tw] OR "Injury" [tw] OR "Injury and Wounds" [tw] OR "Laceration" [tw] OR "Multiple Injuries" [tw] OR "Multiple Injury" [tw] OR "Multiple Traumas" [tw] OR "Multiple Wound" [tw] OR "Multiple Wounds" [tw] OR "Needlestick Injuries" [tw] OR "Open Fracture" [tw] OR "Open Fractures" [tw] OR "Penetrating Wound" [tw] OR "Penetrating Wounds" [tw] OR "Polytrauma" [tw] OR "Polytraumas" [tw] OR "Radiation Induced Dermatitis" [tw] OR "Radiation Recall Dermatitis" [tw] OR "Radiation Recall Reaction" [tw] OR "Radiation Recall Reactions" [tw] OR "Radiation-Induced Dermatitis" [tw] OR "Radiodermatitides" [tw] OR "Research Related Injuries" [tw] OR "Research-Related Injuries" [tw] OR "Research-Related Injury" [tw] OR "Self Mutilating Behavior" [tw] OR "Self Mutilating Behaviors" [tw] OR "Soft Tissue Injury" [tw] OR "Sports Injuries" [tw] OR "Sports Injury" [tw] OR "Sting" [tw] OR "Stings" [tw] OR "Stings and Bites" [tw] OR "Surgical Incision" [tw] OR "Surgical Incisions" [tw] OR "Surgical Wounds" [tw] OR "Trauma" [tw] OR "Trauma, Multiple" [tw] OR "Trauma, War-Related" [tw] OR "Traumas" [tw] OR "Traumatic Amputation" [tw] OR "Traumatic Amputations" [tw] OR "War Related Injuries" [tw] OR "War Related Trauma" [tw] OR "War-Related Injury" [tw] OR "War-Related Trauma" [tw] OR "Wound" [tw] OR "Wounds" [tw] OR "Wounds and Injury" [tw]	
2	(("Religion"[Mesh]) OR "Spirituality"[Mesh]) OR "Spiritual Therapies"[Mesh] OR "Anthroposophy" [tw] OR "Buddhism" [tw] OR "Catholicism" [tw] OR "Christian Science" [tw] OR "Christianity" [tw] OR "Church of Jesus Christ of Latter-day Saints" [tw] OR "Confucianism" [tw] OR "Eastern Orthodoxy" [tw] OR "Ethic, Religious" [tw] OR "Exorcism" [tw] OR "Exorcisms" [tw] OR "Faith Healing" [tw] OR "Hinduism" [tw] OR "Islam" [tw] OR "Jehovah's Witnesses" [tw] OR "Judaism" [tw] OR "Magic" [tw] OR "African Traditional Medicine" [tw] OR "Meditation" [tw] OR "Mental Healing" [tw] OR "Mysticism" [tw] OR "Prayer" [tw] OR "Prayers" [tw] OR "Protestantism" [tw] OR "Radiesthesia" [tw] OR "Religion and Medicine" [tw] OR "Religion and Psychology" [tw] OR "Religion and Science" [tw] OR "Religion and Sex" [tw] OR "Religions" [tw] OR "Religious Belief" [tw] OR "Religious Beliefs" [tw] OR "Religious Ethics" [tw] OR "Religious Missions" [tw] OR "Religious Philosophies" [tw] OR "Saints" [tw] OR "Shamanism" [tw] OR "Spirit Possession" [tw] OR "Spiritual Healing" [tw] OR "Spiritual Healings" [tw] OR "Spiritualism" [tw] OR "Spiritualities " [tw] OR "spirituality" [tw] OR "Spirituality" [tw] OR "Theology" [tw] OR "Therapeutic Touch" [tw] OR "Witchcraft" [tw] OR "Yoga" [tw]	98,428
3	("Wound Healing"[Mesh]) OR "Regeneration"[Mesh] OR heal* [tw] OR regenerat* [tw]	4,619,633
4	#1 AND #2 AND #3	1,397

Embase

**No.**	**Query**	**Results**
#1	'injury'/de OR 'accidental injury'/exp OR 'barotrauma'/exp OR 'bites and stings'/exp OR 'blunt trauma'/exp OR 'crush trauma'/exp OR 'sport injury'/exp OR 'microtrauma'/exp OR 'multiple trauma'/exp OR 'open fracture'/exp OR 'radiation injury'/exp OR 'soft tissue injury'/exp OR 'surgical injury'/exp OR 'wound'/exp OR 'perforation'/exp OR 'accident injuries':ti,ab,kw OR 'accident injury':ti,ab,kw OR 'accidental injury':ti,ab,kw OR 'athletic injury':ti,ab,kw OR 'barotraumas':ti,ab,kw OR 'bite':ti,ab,kw OR 'bites':ti,ab,kw OR 'blast injuries':ti,ab,kw OR 'cold injuries':ti,ab,kw OR 'compound fracture':ti,ab,kw OR 'compound fractures':ti,ab,kw OR 'decapitation':ti,ab,kw OR 'degloving injuries':ti,ab,kw OR 'frostbite':ti,ab,kw OR 'injuries':ti,ab,kw OR 'injuries and wounds':ti,ab,kw OR 'injury':ti,ab,kw OR 'injury and wounds':ti,ab,kw OR 'laceration':ti,ab,kw OR 'multiple injuries':ti,ab,kw OR 'multiple injury':ti,ab,kw OR 'multiple traumas':ti,ab,kw OR 'multiple wound':ti,ab,kw OR 'multiple	2147642

	wounds':ti,ab,kw OR 'needlestick injuries':ti,ab,kw OR 'open fracture':ti,ab,kw OR 'open fractures':ti,ab,kw OR 'penetrating wound':ti,ab,kw OR 'penetrating wounds':ti,ab,kw OR 'polytrauma':ti,ab,kw OR 'polytraumas':ti,ab,kw OR 'radiation induced dermatitis':ti,ab,kw OR 'radiation recall dermatitis':ti,ab,kw OR 'radiation recall reaction':ti,ab,kw OR 'radiation recall reactions':ti,ab,kw OR 'radiation-induced dermatitis':ti,ab,kw OR 'radiodermatitides':ti,ab,kw OR 'research related injuries':ti,ab,kw OR 'research-related injuries':ti,ab,kw OR 'research-related injury':ti,ab,kw OR 'self mutilating behavior':ti,ab,kw OR 'self mutilating behaviors':ti,ab,kw OR 'soft tissue injury':ti,ab,kw OR 'sports injuries':ti,ab,kw OR 'sports injury':ti,ab,kw OR 'sting':ti,ab,kw OR 'stings':ti,ab,kw OR 'stings and bites':ti,ab,kw OR 'surgical incision':ti,ab,kw OR 'surgical incisions':ti,ab,kw OR 'surgical wounds':ti,ab,kw OR 'trauma':ti,ab,kw OR 'trauma, multiple':ti,ab,kw OR 'trauma, war-related':ti,ab,kw OR 'traumas':ti,ab,kw OR 'traumatic amputation':ti,ab,kw OR 'traumatic amputations':ti,ab,kw OR 'war related injuries':ti,ab,kw OR 'war related trauma':ti,ab,kw OR 'war-related injury':ti,ab,kw OR 'war-related trauma':ti,ab,kw OR 'wound':ti,ab,kw OR 'wounds':ti,ab,kw OR 'wounds and injury':ti,ab,kw	
#2	'religion'/exp OR 'spirituality'/exp OR 'spiritual therapies'/exp OR anthroposophy:ti,ab,kw OR buddhism:ti,ab,kw OR catholicism:ti,ab,kw OR 'christian science':ti,ab,kw OR christianity:ti,ab,kw OR 'church of jesus christ of latter-day saints':ti,ab,kw OR confucianism:ti,ab,kw OR 'eastern orthodoxy':ti,ab,kw OR 'religious ethic':ti,ab,kw OR exorcism:ti,ab,kw OR exorcisms:ti,ab,kw OR 'faith healing':ti,ab,kw OR hinduism:ti,ab,kw OR islam:ti,ab,kw OR 'jehovahs witnesses':ti,ab,kw OR judaism:ti,ab,kw OR magic:ti,ab,kw OR 'african traditional medicine':ti,ab,kw OR meditation:ti,ab,kw OR 'mental healing':ti,ab,kw OR mysticism:ti,ab,kw OR prayer:ti,ab,kw OR prayers:ti,ab,kw OR protestantism:ti,ab,kw OR radiesthesia:ti,ab,kw OR 'religion':ti,ab,kw OR religions:ti,ab,kw OR 'religious belief':ti,ab,kw OR 'religious beliefs':ti,ab,kw OR 'religious ethics':ti,ab,kw OR 'religious missions':ti,ab,kw OR 'religious philosophies':ti,ab,kw OR saints:ti,ab,kw OR shamanism:ti,ab,kw OR 'spirit possession':ti,ab,kw OR 'spiritual healing':ti,ab,kw OR 'spiritual healings':ti,ab,kw OR spiritualism:ti,ab,kw OR spiritualities:ti,ab,kw OR spirituality:ti,ab,kw OR theology:ti,ab,kw OR 'therapeutic touch':ti,ab,kw OR witchcraft:ti,ab,kw OR yoga:ti,ab,kw	118079
#3	'wound healing'/exp OR 'regeneration'/exp OR heal*:ti,ab,kw OR regenerat*:ti,ab,kw	5051635
#4	#1 AND #2 AND #3	1719

Cochrane

Search Name:

Date Run: 03/11/2021 10:26:43

Comment:

ID	Search	Hits
#1	((((((((((((((([mh ^"Wounds and Injuries"]) OR [mh "Accidental Injuries"]) OR [mh "Athletic Injuries"]) OR [mh Barotrauma]) OR [mh "Bites and Stings"]) OR [mh "Cold Injury"]) OR [mh "Fractures, Open"]) OR [mh Frostbite]) OR [mh Lacerations]) OR [mh "Microtrauma, Physical"]) OR [mh "Multiple Trauma"]) OR [mh Radiodermatitis]) OR [mh "Self Mutilation"]) OR [mh "Soft Tissue Injuries"]) OR [mh "Surgical Wound"]) OR [mh "War-Related Injuries"]) OR [mh "Wounds, Penetrating"] OR "Accident Injuries":ti,ab,kw OR "Accident Injury":ti,ab,kw OR "Accidental Injury":ti,ab,kw OR "Athletic Injury":ti,ab,kw OR Barotraumas:ti,ab,kw OR Bite:ti,ab,kw OR Bites:ti,ab,kw OR "Blast injuries":ti,ab,kw OR "Cold Injuries":ti,ab,kw OR "Compound Fracture":ti,ab,kw OR "Compound Fractures":ti,ab,kw OR Decapitation:ti,ab,kw OR "Degloving injuries":ti,ab,kw OR Frostbite:ti,ab,kw OR Injuries:ti,ab,kw OR "Injuries and Wounds":ti,ab,kw OR Injury:ti,ab,kw OR "Injury and Wounds":ti,ab,kw OR	101801

	Laceration:ti,ab,kw OR "Multiple Injuries":ti,ab,kw OR "Multiple Injury":ti,ab,kw OR "Multiple Traumas":ti,ab,kw OR "Multiple Wound":ti,ab,kw OR "Multiple Wounds":ti,ab,kw OR "Needlestick Injuries":ti,ab,kw OR "Open Fracture":ti,ab,kw OR "Open Fractures":ti,ab,kw OR "Penetrating Wound":ti,ab,kw OR "Penetrating Wounds":ti,ab,kw OR Polytrauma:ti,ab,kw OR Polytraumas:ti,ab,kw OR "Radiation Induced Dermatitis":ti,ab,kw OR "Radiation Recall Dermatitis":ti,ab,kw OR "Radiation Recall Reaction":ti,ab,kw OR "Radiation Recall Reactions":ti,ab,kw OR "Radiation-Induced Dermatitis":ti,ab,kw OR Radiodermatitides:ti,ab,kw OR "Research Related Injuries":ti,ab,kw OR "Research-Related Injuries":ti,ab,kw OR "Research-Related Injury":ti,ab,kw OR "Self Mutilating Behavior":ti,ab,kw OR "Self Mutilating Behaviors":ti,ab,kw OR "Soft Tissue Injury":ti,ab,kw OR "Sports Injuries":ti,ab,kw OR "Sports Injury":ti,ab,kw OR Sting:ti,ab,kw OR Stings:ti,ab,kw OR "Stings and Bites":ti,ab,kw OR "Surgical Incision":ti,ab,kw OR "Surgical Incisions":ti,ab,kw OR "Surgical Wounds":ti,ab,kw OR Trauma:ti,ab,kw OR "Trauma, Multiple":ti,ab,kw OR "Trauma, War-Related":ti,ab,kw OR Traumas:ti,ab,kw OR "Traumatic Amputation":ti,ab,kw OR "Traumatic Amputations":ti,ab,kw OR "War Related Injuries":ti,ab,kw OR "War Related Trauma":ti,ab,kw OR "War-Related Injury":ti,ab,kw OR "War-Related Trauma":ti,ab,kw OR Wound:ti,ab,kw OR Wounds:ti,ab,kw OR "Wounds and Injury":ti,ab,kw	
#2	(([mh Religion]) OR [mh Spirituality]) OR [mh "Spiritual Therapies"] OR Anthroposophy:ti,ab,kw OR Buddhism:ti,ab,kw OR Catholicism:ti,ab,kw OR "Christian Science":ti,ab,kw OR Christianity:ti,ab,kw OR "Church of Jesus Christ of Latter-day Saints":ti,ab,kw OR Confucianism:ti,ab,kw OR "Eastern Orthodoxy":ti,ab,kw OR "Ethic, Religious":ti,ab,kw OR Exorcism:ti,ab,kw OR Exorcisms:ti,ab,kw OR "Faith Healing":ti,ab,kw OR Hinduism:ti,ab,kw OR Islam:ti,ab,kw OR "Jehovah's Witnesses":ti,ab,kw OR Judaism:ti,ab,kw OR Magic:ti,ab,kw OR "African Traditional Medicine":ti,ab,kw OR Meditation:ti,ab,kw OR "Mental Healing":ti,ab,kw OR Mysticism:ti,ab,kw OR Prayer:ti,ab,kw OR Prayers:ti,ab,kw OR Protestantism:ti,ab,kw OR Radiesthesia:ti,ab,kw OR "Religion and Medicine":ti,ab,kw OR "Religion and Psychology":ti,ab,kw OR "Religion and Science":ti,ab,kw OR "Religion and Sex":ti,ab,kw OR Religions:ti,ab,kw OR "Religious Belief":ti,ab,kw OR "Religious Beliefs":ti,ab,kw OR "Religious Ethics":ti,ab,kw OR "Religious Missions":ti,ab,kw OR "Religious Philosophies":ti,ab,kw OR Saints:ti,ab,kw OR Shamanism:ti,ab,kw OR "Spirit Possession":ti,ab,kw OR "Spiritual Healing":ti,ab,kw OR "Spiritual Healings":ti,ab,kw OR Spiritualism:ti,ab,kw OR Spiritualities:ti,ab,kw OR spirituality:ti,ab,kw OR Spirituality:ti,ab,kw OR Theology:ti,ab,kw OR "Therapeutic Touch":ti,ab,kw OR Witchcraft:ti,ab,kw OR Yoga:ti,ab,kw	8352
#3	([mh "Wound Healing"]) OR [mh Regeneration] OR heal*:ti,ab,kw OR regenerat*:ti,ab,kw	418884
#4	#1 AND #2 AND #3	200

Web of Science

((TS=("Wounds and Injuries" OR "Accidental Injuries" OR "Athletic Injuries" OR Barotrauma OR "Bites and Stings" OR "Cold Injury" OR "Open Fractures" OR Frostbite OR Lacerations OR "Physical Microtrauma" OR "Multiple Trauma" OR Radiodermatitis OR "Self Mutilation" OR "Soft Tissue Injuries" OR "Surgical Wound" OR "War-Related Injuries" OR " Penetrating Wounds" OR "Accident Injuries" OR "Accident Injury" OR "Accidental Injury" OR "Athletic Injury" OR barotrauma OR Bite OR Bites OR "Blast injuries" OR "Cold Injuries" OR "Compound Fracture" OR "Compound Fractures" OR Decapitation OR "Degloving injuries" OR Frostbite OR Injuries OR "Injuries and Wounds" OR Injury OR "Injury and Wounds" OR Laceration OR "Multiple Injuries" OR "Multiple Injury" OR "Multiple Traumas" OR "Multiple Wound" OR "Multiple Wounds" OR "Needlestick Injuries" OR "Open Fracture" OR "Open Fractures" OR "Penetrating

Wound" OR "Penetrating Wounds" OR Polytrauma OR polytrauma OR "Radiation Induced Dermatitis" OR "Radiation Recall Dermatitis" OR "Radiation Recall Reaction" OR "Radiation Recall Reactions" OR "Radiation-Induced Dermatitis" OR radiodermatitis OR "Research Related Injuries" OR "Research-Related Injuries" OR "Research-Related Injury" OR "Self Mutilating Behavior" OR "Self Mutilating Behaviors" OR "Soft Tissue Injury" OR "Sports Injuries" OR "Sports Injury" OR Sting OR Stings OR "Stings and Bites" OR "Surgical Incision" OR "Surgical Incisions" OR "Surgical Wounds" OR Trauma OR "Trauma, Multiple" OR "Trauma, War-Related" OR Traumas OR "Traumatic Amputation" OR "Traumatic Amputations" OR "War Related Injuries" OR "War Related Trauma" OR "War-Related Injury" OR "War-Related Trauma" OR Wound OR Wounds OR "Wounds and Injury"))

AND

TS=(Religion OR Spirituality OR "Spiritual Therapies" OR Anthroposophy OR Buddhism OR Catholicism OR "Christian Science" OR Christianity OR "Church of Jesus Christ of Latter-day Saints" OR Confucianism OR "Eastern Orthodoxy" OR "Ethic, Religious" OR Exorcism OR Exorcisms OR "Faith Healing" OR Hinduism OR Islam OR "Jehovah's Witnesses" OR Judaism OR Magic OR "African Traditional Medicine" OR Meditation OR "Mental Healing" OR Mysticism OR Prayer OR Prayers OR Protestantism OR radiesthesie OR "Religion″ OR Religions OR "Religious Belief" OR "Religious Beliefs" OR "Religious Ethics" OR "Religious Missions" OR "Religious Philosophies" OR Saints OR Shamanism OR "Spirit Possession" OR "Spiritual Healing" OR "Spiritual Healings" OR Spiritualism OR Spiritualities OR spirituality OR Spirituality OR Theology OR "Therapeutic Touch" OR Witchcraft OR Yoga))

AND

TS=("wound healing" OR regenerat* OR heal*)

Searched 11/2/2021

1745 results

Scopus

(TITLE-ABS-KEY (religion OR spirituality OR "Spiritual Therapies" OR anthroposophy OR buddhism OR catholicism OR "Christian Science" OR christianity OR "Church of Jesus Christ of Latter-day Saints" OR confucianism OR "Eastern Orthodoxy" OR "religious ethic" OR exorcism OR exorcisms OR "Faith Healing" OR hinduism OR islam OR "Jehovahs Witnesses" OR judaism OR magic OR "African Traditional Medicine" OR meditation OR "Mental Healing" OR mysticism OR prayer OR prayers OR protestantism OR radiesthesia OR "Religion" OR religions OR "Religious Belief" OR "Religious Beliefs" OR "Religious Ethics" OR "Religious Missions" OR "Religious Philosophies" OR saints OR shamanism OR "Spirit Possession" OR "Spiritual Healing" OR "Spiritual Healings" OR spiritualism OR spiritualities OR spirituality OR spirituality OR theology OR "Therapeutic Touch" OR witchcraft OR yoga) AND TITLE-ABS-KEY ("Wounds and Injuries" OR "Accidental Injuries" OR "Athletic Injuries" OR barotrauma OR "Bites and Stings" OR "Cold Injury" OR "Open Fractures" OR frostbite OR lacerations OR "Physical Microtrauma" OR "Multiple Trauma" OR radiodermatitis OR "Self Mutilation" OR "Soft Tissue Injuries" OR "Surgical Wound" OR "War-Related Injuries" OR " Penetrating Wounds" OR "Accident Injuries" OR "Accident Injury" OR "Accidental Injury" OR "Athletic Injury" OR barotrauma OR bite OR bites OR "Blast injuries" OR "Cold Injuries" OR "Compound Fracture" OR "Compound Fractures" OR decapitation OR "Degloving injuries" OR frostbite OR injuries OR "Injuries and Wounds" OR injury OR "Injury and Wounds" OR laceration OR "Multiple Injuries" OR "Multiple Injury" OR "Multiple Traumas" OR "Multiple Wound" OR "Multiple Wounds" OR "Needlestick Injuries" OR "Open Fracture" OR "Open Fractures" OR "Penetrating Wound" OR "Penetrating Wounds" OR polytrauma OR polytrauma OR "Radiation Induced Dermatitis" OR

"Radiation Recall Dermatitis" OR "Radiation Recall Reaction" OR "Radiation Recall Reactions" OR "Radiation-Induced Dermatitis" OR radiodermatitis OR "Research Related Injuries" OR "Research-Related Injuries" OR "Research-Related Injury" OR "Self Mutilating Behavior" OR "Self Mutilating Behaviors" OR "Soft Tissue Injury" OR "Sports Injuries" OR "Sports Injury" OR sting OR stings OR "Stings and Bites" OR "Surgical Incision" OR "Surgical Incisions" OR "Surgical Wounds" OR trauma OR "Trauma, Multiple" OR "Trauma, War-Related" OR traumas OR "Traumatic Amputation" OR "Traumatic Amputations" OR "War Related Injuries" OR "War Related Trauma" OR "War-Related Injury" OR "War-Related Trauma" OR wound OR wounds OR "Wounds and Injury") AND TITLE-ABS-KEY ("wound healing" OR heal* OR regenerat*))
3079 results

3. Search Summary 3.

Topic	Spirituality and Wound Healing and Critical Care
Reference Manager	
Timeline	
Restrictions or Limitations	
Key Articles	
Databases	PubMed, Embase, Cochrane, Web of Science, Scopus
Date Run	November 2, 2021
Total Number of Results	2578
Number of Duplicates Removed	88
Remaining Number of Results	1689
Search Prepared By	Stella M. Seal, MLS

PubMed

Search number	Query	Results
1	(("Religion"[Mesh]) OR "Spirituality"[Mesh]) OR "Spiritual Therapies"[Mesh] OR "Anthroposophy" [tw] OR "Buddhism" [tw] OR "Catholicism" [tw] OR "Christian Science" [tw] OR "Christianity" [tw] OR "Church of Jesus Christ of Latter-day Saints" [tw] OR "Confucianism" [tw] OR "Eastern Orthodoxy" [tw] OR "Ethic, Religious" [tw] OR "Exorcism" [tw] OR "Exorcisms" [tw] OR "Faith Healing" [tw] OR "Hinduism" [tw] OR "Islam" [tw] OR "Jehovah's Witnesses" [tw] OR "Judaism" [tw] OR "Magic" [tw] OR "African Traditional Medicine" [tw] OR "Meditation" [tw] OR "Mental Healing" [tw] OR "Mysticism" [tw] OR "Prayer" [tw] OR "Prayers" [tw] OR "Protestantism" [tw] OR "Radiesthesia" [tw] OR "Religion and Medicine" [tw] OR "Religion and Psychology" [tw] OR "Religion and Science" [tw] OR "Religion and Sex" [tw] OR "Religions" [tw] OR "Religious Belief" [tw] OR "Religious Beliefs" [tw] OR "Religious Ethics" [tw] OR "Religious Missions" [tw] OR "Religious Philosophies" [tw] OR "Saints" [tw] OR "Shamanism" [tw] OR "Spirit Possession" [tw] OR "Spiritual Healing" [tw] OR "Spiritual Healings" [tw] OR "Spiritualism" [tw] OR "Spiritualities " [tw] OR "spirituality" [tw] OR "Spirituality" [tw] OR "Theology" [tw] OR "Therapeutic Touch" [tw] OR "Witchcraft" [tw] OR "Yoga" [tw]	98,428
2	("Wound Healing"[Mesh]) OR "Regeneration"[Mesh] OR heal* [tw] OR regenerat* [tw]	4,619,633
3	"Critical Care Outcomes"[Mesh] OR "Critical Care"[Mesh] OR "critical care" [tw] OR "intensive care" [tw]	241,660
4	#1 AND #2 AND #3	414

Embase

No.	Query	Results
#1	'religion'/exp OR 'spirituality'/exp OR 'spiritual therapies'/exp OR anthroposophy:ti,ab,kw OR buddhism:ti,ab,kw OR catholicism:ti,ab,kw OR 'christian science':ti,ab,kw OR christianity:ti,ab,kw OR 'church of jesus christ of latter-day saints':ti,ab,kw OR confucianism:ti,ab,kw OR 'eastern orthodoxy':ti,ab,kw OR 'religious ethic':ti,ab,kw OR exorcism:ti,ab,kw OR exorcisms:ti,ab,kw OR 'faith healing':ti,ab,kw OR hinduism:ti,ab,kw OR islam:ti,ab,kw OR 'jehovahs witnesses':ti,ab,kw OR judaism:ti,ab,kw OR magic:ti,ab,kw OR 'african traditional medicine':ti,ab,kw OR meditation:ti,ab,kw OR 'mental healing':ti,ab,kw OR mysticism:ti,ab,kw OR prayer:ti,ab,kw OR prayers:ti,ab,kw OR protestantism:ti,ab,kw OR radiesthesia:ti,ab,kw OR 'religion':ti,ab,kw OR religions:ti,ab,kw OR 'religious belief':ti,ab,kw OR 'religious beliefs':ti,ab,kw OR 'religious ethics':ti,ab,kw OR 'religious missions':ti,ab,kw OR 'religious philosophies':ti,ab,kw OR saints:ti,ab,kw OR shamanism:ti,ab,kw OR 'spirit possession':ti,ab,kw OR 'spiritual healing':ti,ab,kw OR 'spiritual healings':ti,ab,kw OR spiritualism:ti,ab,kw OR spiritualities:ti,ab,kw OR spirituality:ti,ab,kw OR theology:ti,ab,kw OR 'therapeutic touch':ti,ab,kw OR witchcraft:ti,ab,kw OR yoga:ti,ab,kw	118079
#2	'wound healing'/exp OR 'regeneration'/exp OR heal*:ti,ab,kw OR regenerat*:ti,ab,kw	5051635
#3	'intensive care'/exp OR 'critical care outcome'/exp OR (((critical OR intensive) NEAR/3 care):ti,ab,kw)	950965
#4	#1 AND #2 AND #3	787

Cochrane

Search Name:

Date Run: 03/11/2021 10:37:24

Comment:

ID	Search	Hits
#1	(([mh Religion]) OR [mh Spirituality]) OR [mh "Spiritual Therapies"] OR Anthroposophy:ti,ab,kw OR Buddhism:ti,ab,kw OR Catholicism:ti,ab,kw OR "Christian Science":ti,ab,kw OR Christianity:ti,ab,kw OR "Church of Jesus Christ of Latter-day Saints":ti,ab,kw OR Confucianism:ti,ab,kw OR "Eastern Orthodoxy":ti,ab,kw OR "Ethic, Religious":ti,ab,kw OR Exorcism:ti,ab,kw OR Exorcisms:ti,ab,kw OR "Faith Healing":ti,ab,kw OR Hinduism:ti,ab,kw OR Islam:ti,ab,kw OR "Jehovah's Witnesses":ti,ab,kw OR Judaism:ti,ab,kw OR Magic:ti,ab,kw OR "African Traditional Medicine":ti,ab,kw OR Meditation:ti,ab,kw OR "Mental Healing":ti,ab,kw OR Mysticism:ti,ab,kw OR Prayer:ti,ab,kw OR Prayers:ti,ab,kw OR Protestantism:ti,ab,kw OR Radiesthesia:ti,ab,kw OR "Religion and Medicine":ti,ab,kw OR "Religion and Psychology":ti,ab,kw OR "Religion and Science":ti,ab,kw OR "Religion and Sex":ti,ab,kw OR Religions:ti,ab,kw OR "Religious Belief":ti,ab,kw OR "Religious Beliefs":ti,ab,kw OR "Religious Ethics":ti,ab,kw OR "Religious Missions":ti,ab,kw OR "Religious Philosophies":ti,ab,kw OR Saints:ti,ab,kw OR Shamanism:ti,ab,kw OR "Spirit Possession":ti,ab,kw OR "Spiritual Healing":ti,ab,kw OR "Spiritual Healings":ti,ab,kw OR Spiritualism:ti,ab,kw OR Spiritualities:ti,ab,kw OR spirituality:ti,ab,kw OR Spirituality:ti,ab,kw OR Theology:ti,ab,kw OR "Therapeutic Touch":ti,ab,kw OR Witchcraft:ti,ab,kw OR Yoga:ti,ab,kw	8352
#2	([mh "Wound Healing"]) OR [mh Regeneration] OR heal*:ti,ab,kw OR regenerat*:ti,ab,kw	418884
#3	([MH "critical care outcomes"]) OR [mh "critical care"] OR ((critical OR intensive) NEAR/3 care)	50824

#4	#1 AND #2 AND #3	56

Web of Science

TS=(Religion OR Spirituality OR "Spiritual Therapies" OR Anthroposophy OR Buddhism OR Catholicism OR "Christian Science" OR Christianity OR "Church of Jesus Christ of Latter-day Saints" OR Confucianism OR "Eastern Orthodoxy" OR "Ethic, Religious" OR Exorcism OR Exorcisms OR "Faith Healing" OR Hinduism OR Islam OR "Jehovah's Witnesses" OR Judaism OR Magic OR "African Traditional Medicine" OR Meditation OR "Mental Healing" OR Mysticism OR Prayer OR Prayers OR Protestantism OR radiesthesie OR "Religion″ OR Religions OR "Religious Belief" OR "Religious Beliefs" OR "Religious Ethics" OR "Religious Missions" OR "Religious Philosophies" OR Saints OR Shamanism OR "Spirit Possession" OR "Spiritual Healing" OR "Spiritual Healings" OR Spiritualism OR Spiritualities OR spirituality OR Spirituality OR Theology OR "Therapeutic Touch" OR Witchcraft OR Yoga)

AND

TS=("wound healing" OR regenerat* OR heal*)

AND

TS=((critical OR intensive) NEAR/3 care)

Searched 11/2/2021

303 results

Scopus

(TITLE-ABS-KEY (religion OR spirituality OR "Spiritual Therapies" OR anthroposophy OR buddhism OR catholicism OR "Christian Science" OR christianity OR "Church of Jesus Christ of Latter-day Saints" OR confucianism OR "Eastern Orthodoxy" OR "religious ethic" OR exorcism OR exorcisms OR "Faith Healing" OR hinduism OR islam OR "Jehovahs Witnesses" OR judaism OR magic OR "African Traditional Medicine" OR meditation OR "Mental Healing" OR mysticism OR prayer OR prayers OR protestantism OR radiesthesia OR "Religion" OR religions OR "Religious Belief" OR "Religious Beliefs" OR "Religious Ethics" OR "Religious Missions" OR "Religious Philosophies" OR saints OR shamanism OR "Spirit Possession" OR "Spiritual Healing" OR "Spiritual Healings" OR spiritualism OR spiritualities OR spirituality OR spirituality OR theology OR "Therapeutic Touch" OR witchcraft OR yoga) AND TITLE-ABS-KEY ((critical W/3 care) OR (intensive W/3 care)) AND TITLE-ABS-KEY ("wound healing" OR heal* OR regenerat*))

1018 results
